# Supplementary material for: Transiently elevated expression of different forms of brain-derived neurotrophic factor in the neonatal medial prefrontal cortex affected anxiety and depressive-like behaviors in adolescence
Source: PeerJ. 2024 Nov 27;12:e18465. doi: 10.7717/peerj.18465 (PMC11608019; doi:10.7717/peerj.18465)
Supplement: Supplemental Information 9 [file peerj-12-18465-s009.pdf]

Transiently elevated expression of different forms of Brain-derived Neurotrophic Factor in the Neonatal Medial Prefrontal Cortex affected Anxiety and Depressive-like behaviors in Adolescence

Dmitriy Lanshakov, Elizaveta Shaburova, Ekaterina Sukhareva, Veta Bulygina, Uliana Drozd, Irina Larionova, Tatiana Gerashchenko, Tatiana Shnaider, Evgeny Denisov and Tatyana Kalinina

Supplementary materials

Table 1: Primers used in the study, name and sequence

Name	Sequence 5'-3'
BDNF_F	CAT ATC GGC CAC CAA AGA CTC G
BDNF_R	CAA CGG CAA CAA ACC ACA AC
AsuNHproBDNF_F	TGA CGC TAG CAG ATC TAT GAC CAT CCT TTT CCT TAC
AsuNH_BDNF_F	TGA CGC TAG CAG ATC TAT GCA CTC CGA CCC CG
BDNFmut_F	CGC AAA CAT GTC TAT GGC TGC CGC AGC GCA CTC CGA CCC CG
BDNFmut_R	CGG GGT CGG AGT GCG CTG CGG CAG CCA TAG ACA TGT TTG CG
SalBDNF_R	TAG ATG GCG CGC CGT CGA CGC GGC CGC CTA TCT TCC CCT TTT AAT GGT

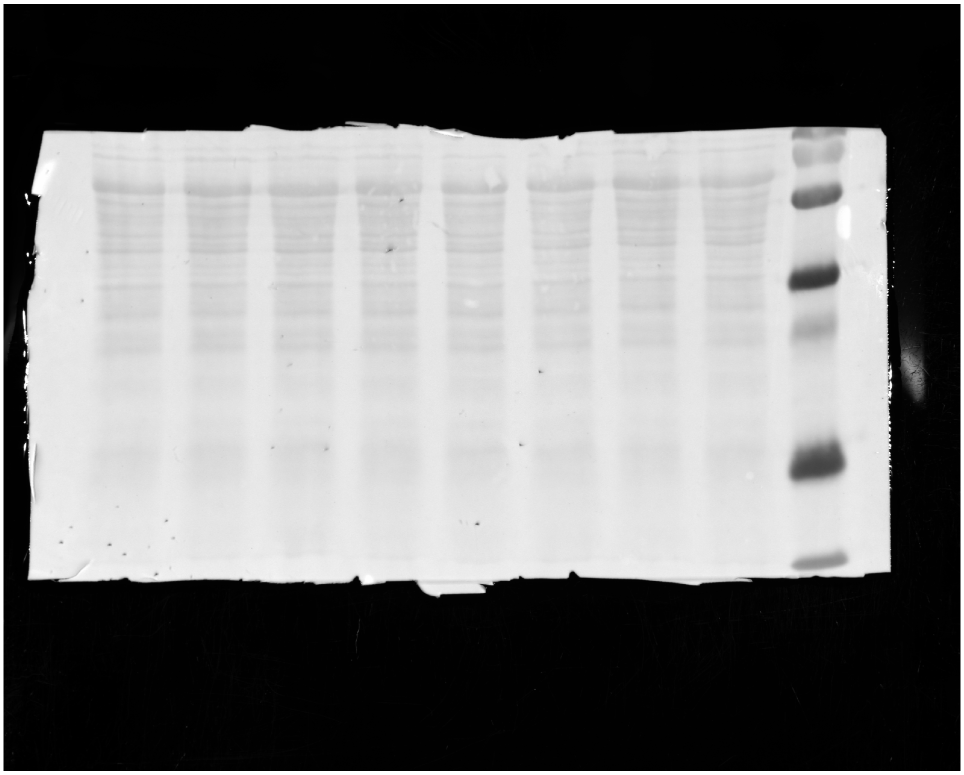

Figure 1: Membrane ponceau staining, protein ladder - PageRuler Plus Prestained Protein Ladder 10 to 250 kDa (26619, Thermo)

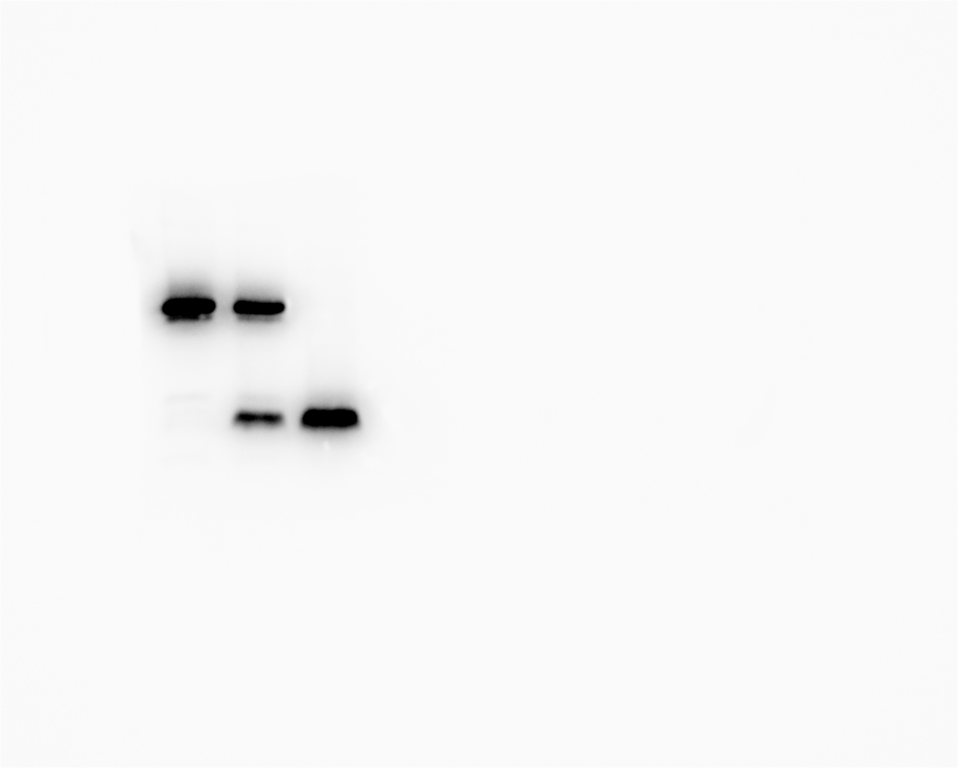

Figure 2: HA-tag immunoblot original image, SAB4300603 Sigma-Aldrich

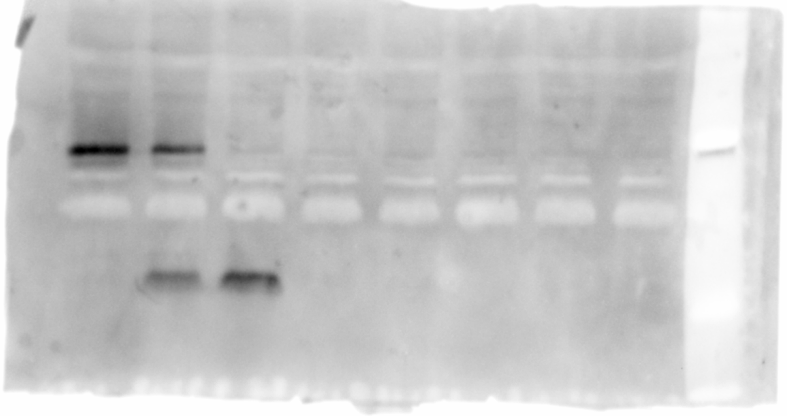

Figure 3: BDNF immunoblot original image, ab6201 Abcam

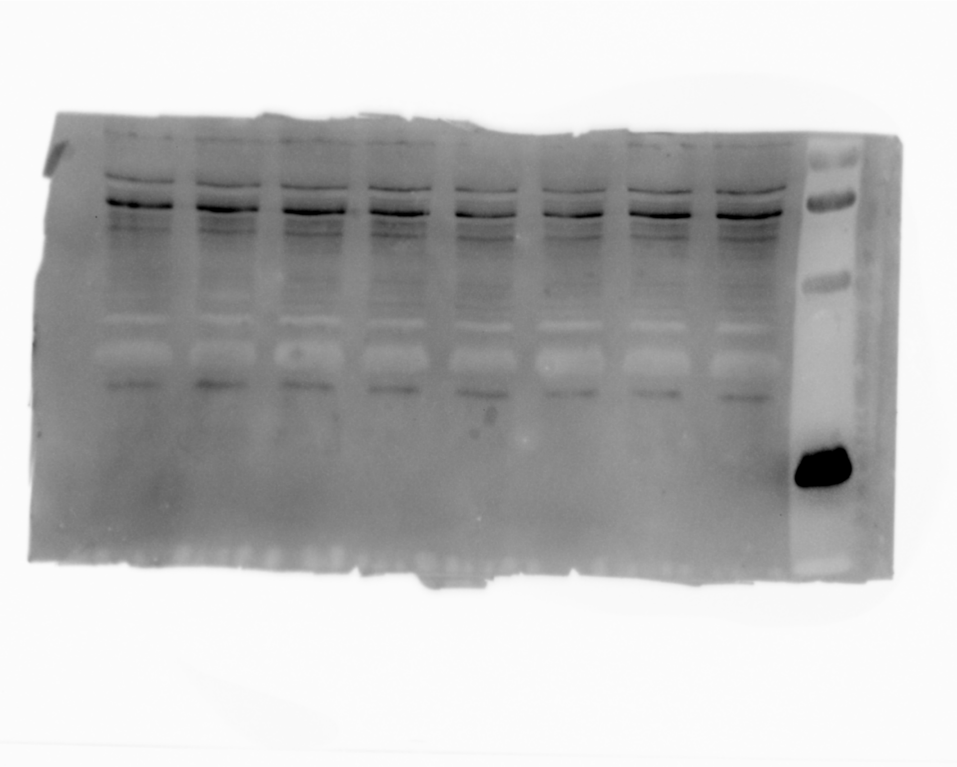

Figure 4: VP16 immunoblot original image, ab4808 Abcam

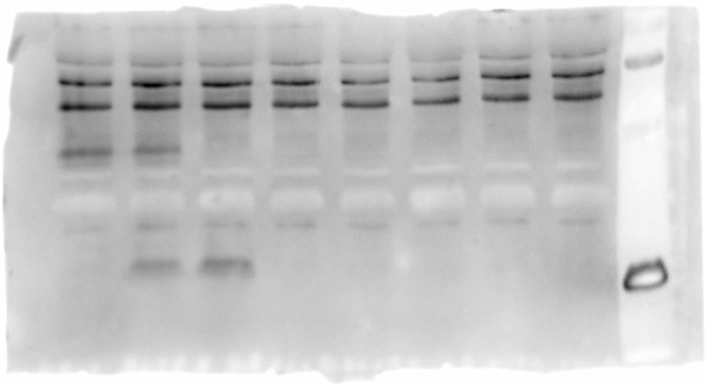

Figure 5: Actin immunoblot on the same membrane without stripping, ab179467 Abcam
